# *Aedes aegypti* has spatially structured and seasonally stable populations in Yogyakarta, Indonesia

**DOI:** 10.1186/s13071-015-1230-6

**Published:** 2015-12-01

**Authors:** Gordana Rašić, Nancy Endersby-Harshman, Warsito Tantowijoyo, Anjali Goundar, Vanessa White, Qiong Yang, Igor Filipović, Petrina Johnson, Ary A. Hoffmann, Eggi Arguni

**Affiliations:** School of BioSciences, Bio21 Institute, The University of Melbourne, Melbourne, VIC 3010 Australia; Eliminate Dengue Project-Yogyakarta, Center for Tropical Medicine, Faculty of Medicine, Universitas Gadjah Mada, Yogyakarta, Indonesia; Eliminate Dengue Project, School of Biological Sciences, Monash University, Melbourne, VIC 3800 Australia

**Keywords:** *Aedes aegypti*, Dengue, Indonesia, Genetic structure, RADseq, Microsatellites, mtDNA

## Abstract

**Background:**

Dengue fever, the most prevalent global arboviral disease, represents an important public health problem in Indonesia. Control of dengue relies on the control of its main vector, the mosquito *Aedes aegypti*, yet nothing is known about the population history and genetic structure of this insect in Indonesia. Our aim was to assess the spatio-temporal population genetic structure of *Ae. aegypti* in Yogyakarta, a densely populated region on Java with common dengue outbreaks.

**Methods:**

We used multiple marker systems (microsatellites, nuclear and mitochondrial genome-wide single nucleotide polymorphisms generated *via* Restriction-site Associated DNA sequencing) to analyze 979 *Ae. aegypti* individuals collected from the Yogyakarta city and the surrounding hamlets during the wet season in 2011 and the following dry season in 2012. We employed individual- and group-based approaches for inferring genetic structure.

**Results:**

We found that *Ae. aegypti* in Yogyakarta has spatially structured and seasonally stable populations. The spatial structuring was significant for the nuclear and mitochondrial markers, while the temporal structuring was non-significant. Nuclear markers identified three main genetic clusters, showing that hamlets have greater genetic isolation from each other and from the inner city sites. However, one hamlet experienced unrestricted mosquito interbreeding with the inner city, forming a single genetic cluster. Genetic distance was poorly correlated with the spatial distance among mosquito samples, suggesting stronger influence of human-assisted gene flow than active mosquito movement on spatial genetic structure. A star-shaped mitochondrial haplotype network and a significant *R*^2^ test statistic (*R*^2^ = 0.0187, *P* = 0.001) support the hypothesis that *Ae. aegypti* in Yogyakarta originated from a small or homogeneous source and has undergone a relatively recent demographic expansion.

**Conclusion:**

We report the first insights into the spatio-temporal genetic structure and the underlying processes in the dengue fever mosquito from Yogyakarta, Indonesia. Our results provide valuable information on the effectiveness of local control measures as well as guidelines for the implementation of novel biocontrol strategies such as release of *Wolbachia*-infected mosquitoes.

**Electronic supplementary material:**

The online version of this article (doi:10.1186/s13071-015-1230-6) contains supplementary material, which is available to authorized users.

## Background

Dengue fever is the most prevalent arboviral disease in the world and Indonesia boasts one of the highest numbers of dengue infections amongst the most endemic nations [1]. Moreover, incidence of a severe disease form known as the dengue hemorrhagic fever has dramatically increased in Indonesia over the past 45 years [2]. A surge in dengue outbreaks is attributed to a combination of factors such as rapid human population growth, migration from rural to urban areas, inadequate basic urban infrastructure and public health measures [3], all of which favor viral transmission by the main vector *Aedes aegypti*. This mosquito has a close association with human habitation, preferentially exploiting domestic environments for its feeding and breeding requirements [4–6].

Given that an effective vaccines or treatments against dengue viruses are not yet available, the control of dengue transmission is mostly dependent on the control of the vector populations [1]. Insecticide applications remain the predominant strategy to control the dengue fever mosquito, but their extensive and inappropriate use has led to a development of insecticide resistance in *Ae. aegypti* populations around the world, including Indonesia [7, 8]. In the last 15 years, a significant advancement has been made in developing and implementing alternative strategies to overcome the limitation of traditional measures and to achieve a sustainable control of vector populations [9]. One such strategy involves the use of the intracellular bacterium *Wolbachia pipientis* that has been artificially transferred into *Ae. aegypti* [10, 11]. *Wolbachia* infection induces a number of pathogen-resistant phenotypes that reduce the mosquito’s vectorial competency [12]. This strategy is based on the releases of *Wolbachia*-infected *Ae. aegypti* into the field to replace the vector competent populations with mosquitoes that have a significantly diminished capacity for pathogen transmission [13–15].

Following the successful *Wolbachia* releases in northern Australia and Vietnam [15, 16], the implementation of this strategy is planned for the region of Yogyakarta (http://www.eliminatedengue.com/indonesia). Given that the establishment and spread of *Wolbachia* infection is influenced by the dynamics of local populations as well as migration among them [17, 18], it is essential to obtain knowledge of the population genetic structure and gene flow in *Ae. aegypti* throughout Yogyakarta.

Apart from information on the strong genome-wide differentiation between a small sample of *Ae. aegypti* from Indonesia and samples from Vietnam, Australia and Brazil [19], there is no knowledge on the population genetic structure in the dengue fever mosquito from Indonesia. Studies from other countries in southeast Asia have generally shown significant population structuring for a range of spatial scales (5 to >2000 km) [20]. However, the level of genetic structuring was highly variable among areas with different levels of urbanization (rural vs. urban) or between seasons (rainy vs. dry) (reviewed in [21]).

Here, our aim was to assess the spatio-temporal population genetic structure of *Ae. aegypti* in the city of Yogyakarta and the surrounding hamlets. This large and densely populated urban environment on the island of Java with an approximate population of 3.4 million people has common dengue outbreaks [22]. For example, an average annual rate of 16.8 dengue cases per 10,000 inhabitants has been reported in Yogyakarta city from 2005 to 2007 (Yogyakarta City Health Office, 2008). We used several types of genetic marker (microsatellites, nuclear and mitochondrial genome-wide single nucleotide polymorphisms) to genotype individuals of *Ae. aegypti* collected during one wet and one dry season. We considered the following questions: 1) what is the level of structuring at the nuclear and mitochondrial genomes among sites in Yogyakarta? 2) Is there a seasonal variation in genetic structuring? 3) Is the level of gene flow different among urban and suburban areas (i.e. Yogyakarta city and hamlets)?

## Methods

### Sampling

**Fig. 1:**
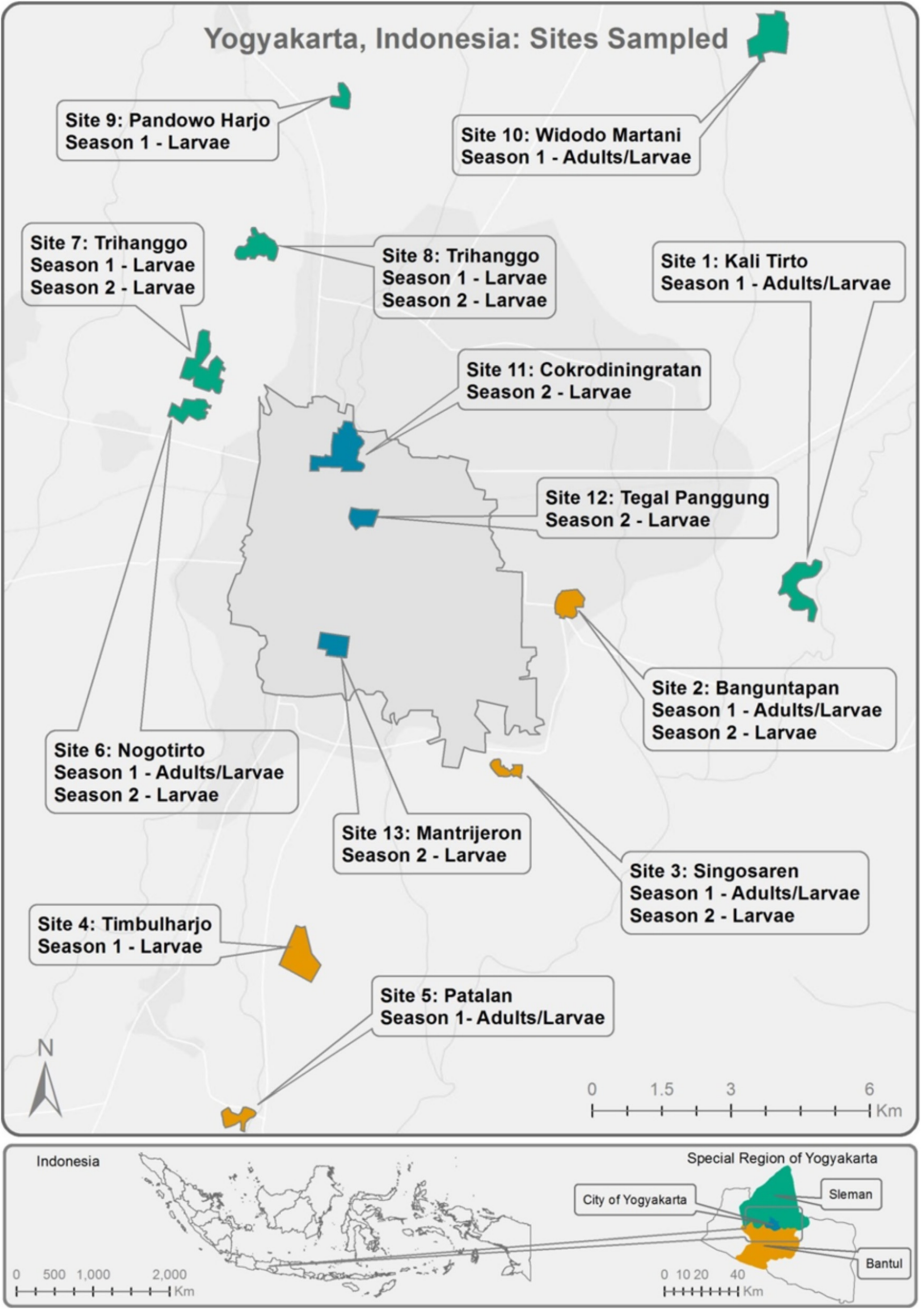
Collection sites in Yogyakarta region in central Java, Indonesia. *Aedes aegypti* adults and larvae were sampled in the wet season in 2011 and in the following dry season in 2012. The inner city area (with sites 11–13) is delineated with a border line

Thirteen collection sites within three districts were established in the Special Region of Yogyakarta for this study (Fig. 1, Table 1). Samples were collected from the districts of Bantul and Sleman between October to December of 2011 as a representation of the wet season and in July of 2012 as a representation of the dry season. The average precipitation in Yogyakarta in July is 40 mm and increases to 90–340 mm in October to December. The sites are separated by areas of agricultural land with many large roads/highways and rivers/canals which are likely to impede mosquito dispersal. Dry season samples also included a collection made in November 2012 from the City of Yogyakarta (Fig. 1, sites 11–13), allowing for the study of mosquito population structure between continuously populated sites without the agricultural lands. The area covered by districts Sleman, Yogyakarta City and Bantul is approximately 1,115.45 km2 with each collection site spanning between 0.1 km^2^ and 1.0 km^2^.

**Table 1:** Indices of population genetic diversity for *Aedes aegypti* in Yogyakarta, Indonesia

			Microsatellites				nuclear SNPs			mtDNA		
Site	Hamlet	Life stage/season	n	Allelic richness	*H*_E_	*F*_IS_	n	*H*_E_	*F*_IS_	n	*π*	*H*d
1	Kali Tirto	L/wet	52	3.99	0.532	0.020	-	-	-	-	-	-
		A/wet	21	3.87	0.506	0.138	-	-	-	-	-	-
2	Banguntapan	L/wet	79	4.54	0.614	0.069	-	-	-	-	-	-
		L/dry	34	5.81	0.597	0.055	9	0.210	0.157	19	0.0009	0.713
		A/wet	27	5.31	0.607	−0.010	-	-	-	-	-	-
3	Singosaren	L/wet	23	4.44	0.560	0.132	-	-	-	-	-	-
		L/dry	36	5.26	0.553	0.169	5	0.209	−0.011	15	0.0013	0.819
		A/wet	30	5.05	0.598	0.083	-	-	-	-	-	-
4	Timbulharjo	L/wet	16	3.81	0.569	0.005	-	-	-	-	-	-
5	Patalan	L/wet	17	4.25	0.549	0.164	-	-	-	-	-	-
		A/wet	23	4.64	0.547	0.035	-	-	-	-	-	-
6	Nogotirto	L/wet	34	4.13	0.583	0.044	10	0.205	0.077	6	0.0044	0.800
		L/dry	37	5.14	0.593	0.073	12	0.207	0.051	9	0.0012	0.694
		A/wet	27	4.30	0.522	0.030	-	-	-	-	-	-
7	Trihanggo	L/wet	46	3.89	0.522	0.055	16	0.214	0.051	10	0.0012	0.533
		L/dry	36	5.17	0.523	0.035	12	0.217	0.107	9	0.0007	0.250
8	Trihanggo	L/wet	30	3.86	0.547	0.085	20	0.227	0.119	8	0.0013	0.464
		L/dry	34	5.47	0.555	0.065	20	0.221	0.143	8	0.0016	0.667
9	Pandowo Harjo	L/wet	10	4.00	0.561	0.135	-	-	-	-	-	-
10	Widodo Martani	L/wet	18	3.82	0.559	0.024	-	-	-	-	-	-
		A/dry	24	3.98	0.548	0.029	-	-	-	-	-	-
11	Cokrodiningratan	L/dry	40	5.54	0.538	−0.005	19	0.226	0.094	9	0.0021	0.972
12	Tegal Panggung	L/dry	40	5.47	0.543	0.039	20	0.227	0.102	13	0.0005	0.641
13	Mantrijeron	L/dry	40	5.44	0.574	0.025	20	0.230	0.127	17	0.0004	0.721

Indices for microsatellites, nuclear-genome wide SNPs and mitochondrial DNA sequences. *n* sample size, *L* larvae, *A* adults, season: wet and dry; *H*_*E*_ expected heterozygosity, *F*_IS_ inbreeding/fixation index, *π* nucleotide diversity, *H*_d_ haplotype diversity

Within each collection site, *Aedes aegypti* larvae and adults were collected from ovitraps and backpack aspirators (BPA) positioned inside or very close to the houses (i.e. front- or backyards). Eggs collected from ovitraps were reared to the third larval instar to facilitate identification of species. A single larva from each ovitrap was used for analysis to minimise sampling of family groups [19, 23, 24], while multiple adults from each house were sampled and analysed.

### Microsatellite typing

Individuals were genotyped at nine microsatellite markers previously described (AC1, AG5, 88AT1, BbA10, BbH08, M201, 12ACG1, 470AG1 and 69TGA1 [25–27]). The loci were separated into three multiplex PCR assays as described in [28]. Microsatellite fragment separation analysis was conducted using the Applied Biosystems 96-capillary 3730xl DNA Analyser and GeneScan™ 500 LIZ® size standard and scored using GeneMarker® Software Version 1.91 [29]. The final microsatellite dataset included: 152 adults and 351 larvae sampled across hamlets in wet season, and 320 larvae sampled from hamlets and inner city during dry season (Table 1).

### SNP typing

Genome-wide single nucleotide polymorphism (SNP) genotyping was done using a customized double-digest RADseq method [30] as described in [19]. 100 ng of genomic DNA from each larva was digested with 10 units of restriction enzymes *NlaIII* and *MluCI* (New England Biolabs, Beverly MA, USA). Illumina adapters with customized barcode sequences (100 pM of P1, 300 pM of P2) were ligated to the genomic fragments using 100 units of T4 ligase at 16 °C overnight (New England Biolabs, Beverly, MA, USA). Size selection of 300–450 bp long fragments was completed with the Pippin Prep protocol (Sage Sciences, Beverly, MA, USA). For the final library enrichment, five repeated PCR reactions with 1 nM standard Illumina primers included the following cycling conditions: 98 °C for 30 s, 12 cycles of 98 °C for 10 s, 60 °C for 30 s, 72 °C for 90 s, and the final elongation at 72 °C for 5 min. The independent reactions were pooled and purified to create a final library. Four libraries were sequenced in four lanes of Illumina HiSeq2000 using the 100 bp paired-end chemistry (SRA Accession Numbers: SRP040064, SRX1425749).

Raw Illumina reads were filtered and trimmed based on a minimum phred score of 20. We used the short read aligner program Bowtie [31] to align the filtered reads to the *Ae. aegypti* nuclear and mitochondrial reference genomes [32, 33]. Because we expected our populations to show substantial genome divergence from the reference strain (i.e. Liverpool strain that originated from a West African population [32]), we allowed for the maximum number of mismatches in the alignment seed. The search algorithm was set to try as hard as possible to find valid alignments when they existed [Bowtie option—tryhard], and only uniquely aligned reads were then analysed with the Stacks pipeline [34]. Default program parameters were used for variant and likelihood-based genotype calling at RAD stacks that had a depth of at least five reads. The final RADseq dataset included 46 larvae collected across three hamlets in the wet season and 117 larvae collected from five hamlets and three inner city locations in the dry season (Table 1, Additional file 1: Table S1). 27 larvae from the wet season had more than 30 % missing data for nuclear SNPs and were excluded from downstream analyses for this marker set.

### Analyses of genetic diversity and structure

#### Nuclear markers

Basic population genetic analyses such as observed and expected heterozygosity and tests for departure from Hardy-Weinberg equilibrium were carried out using GenAlEx v6.5 [35] and Fstat 2.9.3.2 [36]. We used the program FreeNA to test for the presence of null alleles at microsatellite loci that can bias the estimates of within-population diversity and population structure [37].

To estimate the level of spatio-temporal genetic structuring, we performed the hierarchical analysis of molecular variance (AMOVA) and post-hoc pairwise *F*_ST_ comparisons, treating each sampling location as an independent sample nested within season. Statistical significance was determined from 999 permutations using the program GenAlEx v6.5 [35].

Genetic structure was also inferred using the individual-based analysis implemented in the program STRUCTURE v2.3.4 [38, 39], that uses multi-locus genotypes to determine the number of genetic groups (K) and assign individuals to those groups. The parameter set included: admixture model with the information on the sampling location and correlated allele frequencies among populations. The number of burn-ins was 250,000 for the microsatellite datasets and 50,000 for the SNP datasets, followed by as many MCMC steps. Estimations were made for K = 1 to N, where N represents the number of sampling sites in a given season. The most likely number of clusters was determined using the maximum likelihood guideline by Pritchard [40] and the deltaK method by Evanno et al. [41] implemented with the program Structure Harvester [42]. We also used a multivariate method called discriminant analysis of principal components (DAPC) [43] implemented within the R package adegenet [44], to infer the number of genetic groups and the individual assignment to those groups.

Presence of isolation by distance (IBD) [45] between sampling groups was estimated using the matrices of pair-wise linearized *F*_ST_ values and the natural log of pairwise geographic distance between sites. IBD among individuals was tested using the matrix of unweighted squared genetic distances between individuals [46] and the natural log of pairwise geographic distance between individuals. Significance of the correlation between the matrices of genetic and geographic distances was determined using a Mantel test with 999 permutations in GenAlEx v6.5 [35]. The spatial autocorrelation analysis [46] with 999 permutations was executed in the same program. An estimate of effective population size, *N*e, was obtained using the two-sample temporal analysis method [47] as applied in NeEstimator 2.01 [48] for both microsatellite and SNP data at sites 6, 7 and 8. Generations sampled were set at 0 and 10 based on the length of time between the two field collections and estimated 15 generations per year in Yogyakarta. Waples’ method [47] uses moments-based *F* statistics and, in this case, the estimator *F*s [49] was applied as it has been developed to provide unbiased estimates of *Ne* for multiple types of data sets including large panels of SNPs.

#### Mitochondrial markers

The analyses of the number of mitochondrial haplotypes (*N*h), nucleotide diversity (*π*), haplotype diversity (*H*d), as well as the spatio-temporal AMOVA were done in the R package pegas [50]. We used *R*^2^ test [51] for detecting population growth, determining the statistical significance with 1000 simulations of populations under the drift-mutation equilibrium in pegas [50]. The *R*^2^ test has greater power than a classical mismatch distribution test for smaller sample sizes [51]. Haplotype network analysis with the statistical parsimony method was completed with the R package TempNet [52].

## Results

### Nuclear diversity

Allelic richness over nine microsatellite loci within each site ranged between 3.81 to 5.31 in wet season and between 5.14 and 5.81 in dry season (Table 1). Site 2 exhibited the highest allelic richness in both seasons. In general, observed heterozygosity at each sampling location was lower than expected heterozygosity (Table 1) and markers did not exhibit significant linkage disequilibrium.

After removing nuclear RAD tags with more than 30 % of missing data, the final dataset included 3,178 polymorphic tags distributed across 830 scaffolds on all three chromosomes of *Ae. aegypti.* Overall nucleotide diversity in these RAD tags was 0.0023. Because multiple SNPs within the same RAD tag are expected to be in nearly complete linkage disequilibrium, we retained only one SNP per tag. Expected heterozygosity across 3,178 SNPs was similar for all sites, ranging between 0.205 and 0.230, and was somewhat higher than the observed heterozygosity (*F*_IS_ from 0 to 0.157, Table 1).

### Nuclear genetic structure

For both marker systems, hierarchical analysis of molecular variance (AMOVA) indicated strong spatial genetic structure that was temporally stable (Table 2). Three percent of total variation at microsatellite loci was attributed to differences in allele frequencies among sites within a season, while no significant variation could be attributed to changes across seasons. Spatial difference in frequencies of nuclear genome-wide SNPs contributed to nearly seven percent of total variation (Table 2). Pairwise *F*_ST_ comparisons at SNPs revealed that mosquitoes from sites 2 and 6 showed the highest differentiation at within the region (Table 3). Also, adult and larval samples collected within the same hamlet did not show a significant difference in allele frequencies, except at site 6 (Additional file 1: Table S1). Genome-wide SNPs revealed much lower differentiation among city sites than among hamlets, while microsatellite markers did not reveal this pattern (Table 3).

**Table 2:** Hierarchical analysis of molecular variance (AMOVA) with sites nested within seasons

AMOVA Summary					
nuclear SNPs					
Source	df	SS	MS	Est. Var.	%
season	1	2447.806	2447.806	2.882	0.1
site	6	13576.461	1939.494	36.268	6.8
Error	318	160791.500	510.349	510.449	93.1
Total	325	176815.767		549.599	100.0
mtDNA					
Source	df	SS	MS	Est. Var.	%
season	1	3.922	3.922	0.013	0.4
site	6	21.663	3.610	0.095	4.6
Error	131	257.207	1.963	1.963	95.0
Total	138	282.791		2.071	100.0

Datasets include nuclear SNPs and mitochondrial DNA sequences from *Aedes aegypti* larvae from Yogyakarta, Indonesia

**Table 3:**
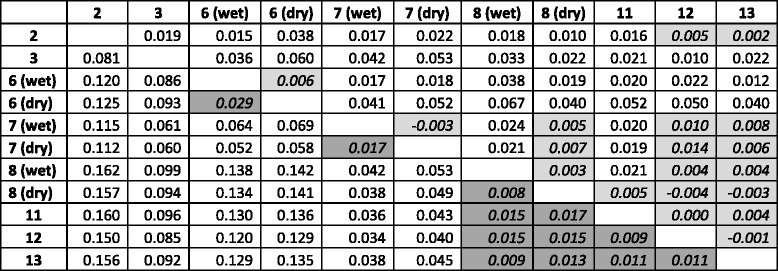
Pair-wise *F*_ST_ values for spatial and temporal samples of *Aedes aegypti* from Yogyakarta, Indonesia*.* Larvae were typed at nuclear SNPs (bellow diagonal) and microsatellite markers (above diagonal). Seasonal samples are designated with a bracket, e.g. sample from site 7 collected in the wet season is labeled as 7 (wet). Non-significant *F*_ST_ values are shaded

STRUCTURE analyses of adult mosquitoes suggested four genetic groups based on the highest likelihood of data, and two groups based on the deltaK method (Additional file 1: Table S2). For the larval samples, both methods indicated that the number of genetic groups was three in the wet season and two in the following dry season (Additional file 1: Table S2).

**Fig. 2:**
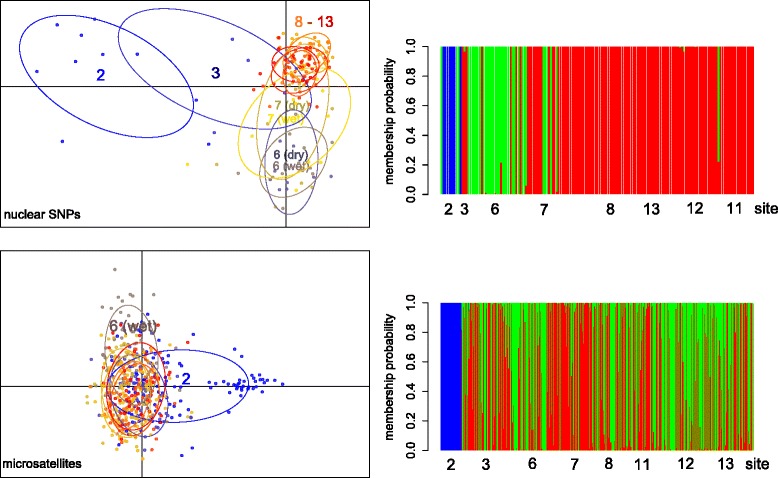
Discriminant analysis of principal components (DAPC) for *Aedes aegypti* from Yogyakarta, Indonesia. Individual larvae collected across hamlets and inner city sites were genotyped at nuclear genome-wide SNPs (upper) and microsatellites (lower). Scatterplots with individuals (dots) from different sites labeled with different colors is on the left. Membership probabilities to the three derived genetic groups for individuals (vertical lines) collected at different sites is on the right

Genome-wide SNPs showed much greater power in distinguishing *Ae. aegypti* individuals from different sites in Yogyakarta than microsatellite markers (Fig. 2) and DAPC without prior information on the sampling location (‘blind DAPC’) revealed three genetic clusters in the SNP dataset. Again, sites 2 and 6 showed greater separation from other sites in the region, while site 8 and inner city cites could not be distinguished. Moreover, temporal stability of genetic structure was demonstrated by the overlap of temporal samples in the PC space (Fig. 2).

**Fig. 3:**
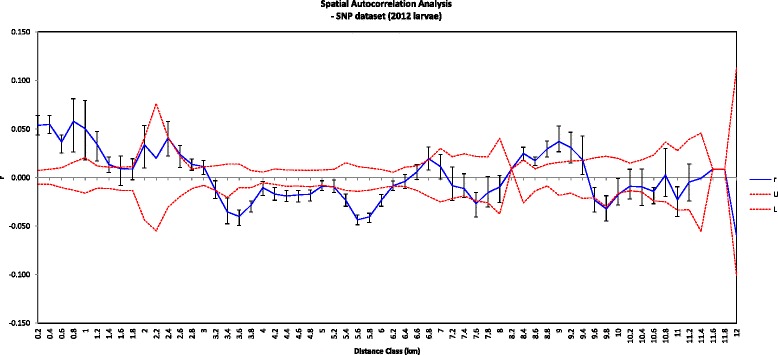
Spatial autocorrelation analysis for *Aedes aegypti* larvae collected in the dry season. Larvae were genotyped at 3,178 SNPs. Upper (U) and lower (L) bounds for the non-significant autocorrelation coefficient (*r*) were determined using 999 permutations

Presence of IBD was highly supported between individual adult mosquitoes (microsatellites: Mantel *r* = 0.106, *P* < 0.01), and individual larvae in the season (microsatellites: Mantel *r* = 0.106, *P* < 0.01; SNPs: Mantel *r* = 0.247, *P* < 0.01). Spatial autocorrelation analysis indicated that greater relatedness between pairs of larvae can be found at distances of 8–9 km, even though it is low for larvae from different sites (i.e. autocorrelation coefficient is significantly negative at distances 3–6 km) (Fig. 3).

Site 6 had the lowest estimated *N*e out of the three sites tested, using either microsatellite or SNP data (Table 4). Tighter estimates of *N*e for the SNP data are in line with the observation that current methods tend to produce intervals that are too narrow when a large number of markers is used [48]. 95 % confidence intervals for *N*e generated from SNP data were 128–144, 265–298 and 689–774, for Sites 6, 7 and 8 respectively (Table 4). For microsatellite data, estimates from the same sites were 212–564, infinite and 449–1303, respectively. Infinite value for the unbiased Ne estimator means that temporal genetic variation can be explained by the sampling error alone, and not by the effects of a finite number of parents [48].

**Table 4:** Temporal estimates of effective population size (*N*e) for *Aedes aegypti* from Yogyakrta, Indonesia

Site	6		7		8	
Marker type	msats	SNPs	msats	SNPs	msats	SNPs
No. of independent alleles	33	2104	28	2172	28	2286
Harmonic mean sample size	35	8	42	12	34	19
*F*s	0.04222	0.16824	0.02079	0.10359	0.03595	0.05988
*F*’	0.01363	0.03676	−0.0032	0.0178	0.00609	0.00684
*N*e	367	136	Infinite	281	820	730
95 % CIs for *N*_e_	212	128	Infinite	264	449	689
	563	144	Infinite	298	1303	773
Jackknife on loci	116	105	964	201	143	483
	Infinite	192	Infinite	464	Infinite	1498

Seasonal mosquito larvae were collected from Sites 6, 7 and 8 in Yogyakarta and typed at microsatellites and nuclear genome-wide SNPs with a minimum allele frequency of 5 %

### Mitochondrial diversity and structure

**Fig. 4:**
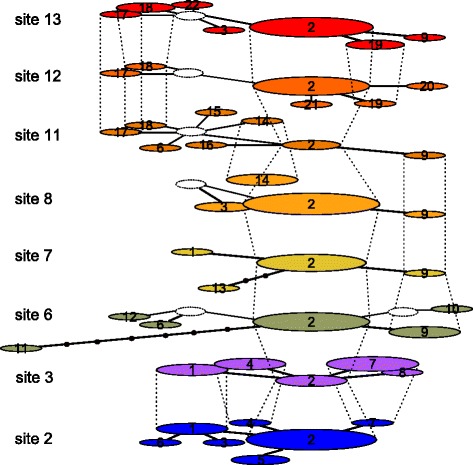
Haplotype networks depicting spatial variation of mitochondrial haplotypes. *Aedes aegypti* samples were collected at 5 hamlets (sites 2,3,6–8) and 3 inner city sites (11–13) in Yogyakarta, Indonesia. Dotted vertical lines connect identical haplotypes found at different sites. Ellipse size corresponds to the number of individuals with a given haplotype. A number within an ellipse denominates the specific haplotype (1–22)

**Fig. 5:**
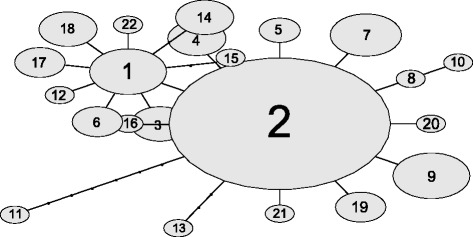
Haplotype network depicting total variation of mitochondrial haplotypes in *Aedes aegypti* from Yogyakarta. A number within an ellipse designates a specific haplotype (1–22), with the ellipse size proportional to the number of individuals with a given haplotype

We found 16 RAD tags in eight mitochondrial genes (*ND2, COXI, COX3, ATP6, ND4-ND6, CYTB*) that were present in more than 80 % of individuals. We first checked for the presence of premature stop codons or heterozygous loci that would suggest paralogous/nuclear copies of mitochondrial sequences [53, 54] and then we concatenated all tags into sequences that were 990 bp long. Additionally, we confirmed that in 14 individuals ddRAD tags had sequences identical to the longer amplicons from the corresponding mitochondrial regions [55]. Nevertheless, it is possible to have some spurious signals originating from the nuclear copies despite the control steps we employed in our study. There were 22 unique haplotypes in the full dataset (combined across sites and seasons), with haplotype 2 being shared among all eight sites, and haplotype 9 among five sites (Fig. 4). Haplotype diversity ranged between 0.250 and 0.972 and nucleotide diversity ranged between 0.0004 and 0.0044 (Table 1, Fig. 4). AMOVA revealed significant spatial structure after accounting for small seasonal changes of the mitochondrial haplotype frequencies (Table 2). The haplotype network for the entire dataset was star-shaped, with one predominant haplotype (haplotype 2) and pair-wise haplotype differences ranging between one and eight base pairs (Fig. 5). The star-shaped haplotype network suggests exponential population growth [56], and this hypothesis was supported with the *R*^2^ test for recent population expansion (*R*^2^ = 0.0187, *P* = 0.001).

## Discussion

Multiple marker systems and temporal sampling of *Aedes aegypti* across Yogyakarta revealed that the dengue fever mosquito has spatially structured and seasonally stable populations in this region of Indonesia. Nuclear markers (genome-wide SNPs and microsatellites) showed that mosquitoes from some hamlets exhibit greater genetic isolation, regardless of their geographic position (e.g. sites 2 and 6). Also, mosquitoes from inner city sites and one hamlet (site 8) seem to interbreed freely, constituting a single genetic cluster. Mitochondrial variation suggests relatively recent population expansion of *Ae. aegypti* in this region.

*Aedes aegypti* is thought to have invaded southeast Asia from the New World as recently as the 20th century [57]. Mitochondrial data available in our study do not allow for identification of the origin of *Ae. aegypti* in Yogyakarta, but the presence of a single haplogroup supports the hypothesis of a small or genetically homogeneous source that underwent population expansion. This is in sharp contrast to *Ae. aegypti* from Rio de Janeiro, Brazil, where several highly divergent mitochondrial lineages have been found in one continuously distributed large population [58]. Our results are consistent with previous reports of much lower genetic diversity in *Ae. aegypti* populations from southeast Asia when compared with those in the New World. For example, Bosio et al. [59] found that the dengue fever mosquito in Thailand harbors mitochondrial diversity that is 2–3 times lower than that in Mexico.

Analyses of nuclear markers suggest stronger influence of human-assisted gene flow than active dispersal in shaping the spatial pattern of genetic structure in *Ae. aegypti* from Yogyakarta. Genetic clustering did not clearly correspond to the spatial distance among sampling locations, resulting in a very weak isolation-by-distance. Generally, hamlets showed greater genetic isolation from each other and from the inner city sites. However, one hamlet (site 8) exhibited unrestricted interbreeding with mosquitoes from the inner city, forming a single genetic cluster. The spatial genetic structure of *Ae. aegypti* in Indonesia parallels the examples from Thailand, where mosquitoes show overall weaker differentiation within urban areas such as Bangkok or Hat Yai than in suburban or rural areas [60, 61]. Exceptions to these common patterns, like the case of high genetic connectivity between some hamlets and the urban center in Yogyakarta, could help identify specific human activities/means of transportation that facilitate extensive long distance gene flow in this mosquito.

A recent entomological study in Yogyakarta found that numbers of adult mosquitoes tended to fluctuate by season in sites 2 and 6, but were stable throughout a year in sites 7 and 8 [62]. We did not detect any significant seasonal changes in genetic structure at site 6, but the estimated effective population size was several times lower than in site 8. Some of the local variation in mosquito numbers may be attributed to differences in rainfall patterns that varied across hamlets [62]. Other factors could include locally varying mosquito control measures, such as removal of breeding habitats and application of insecticides which has been widespread in Indonesia since the 1970s [63]. However, our data suggest that local control measures have had little overall success in reducing mosquito numbers to levels that would cause significant genetic bottlenecks, as seen from a temporally stable genetic structure in populations of *Ae. aegypti* across Yogyakarta. Diminishing efficacy of insecticide-based mosquito control is seen globally due to rapidly evolving resistance to all classes of insecticides [64]. Recent results from bioassays and genetic screening of putative resistance alleles in *Ae. aegypti* demonstrated that pyrethroid insecticides are likely to be losing efficacy in Yogyakarta, urging the need for the employment of resistance management tactics [65]. Our insights into the genetic connectivity of mosquito populations should assist in predicting the spread of the resistance alleles in this region.

Knowledge of the spatio-temporal genetic diversity and structuring in *Ae. aegypti* from Yogyakarta obtained through our study is also useful for implementation of *Wolbachia*–based strategies in this region. Replacement of local mosquito populations with *Wolbachia*¬infected mosquitoes could be more challenging in hamlet 8 and within the city sites due to high migration of uninfected mosquitoes into the release area. This could require a process of “adaptive management”, where an increased rate of releases is undertaken in areas with slower *Wolbachia* invasion [15].

## Conclusion

Our study provides the first insight into the genetic diversity and structure of the dengue fever mosquito, *Aedes aegypti*, in Indonesia where dengue is endemic. Seasonal stability of population genetic structure and diversity suggests limited effectiveness of traditional control measures to reduce numbers of breeding mosquitoes. Finally, knowledge of the genetic connectivity/isolation of mosquitoes in some parts of Yogyakarta will be useful for optimizing the strategy that involves the releases of *Wolbachia*-infected mosquitoes to suppress dengue transmission.

## Additional file

Additional file 1: Table S1. Pairwise *F*_ST_ comparisons between *Aedes aegypti* adults and larvae. Individuals collected in wet season were scored at nine microsatellite loci. **Table S2.** STRUCTURE Harvester results for adult and larval mosquitoes that were scored at nine microsatellite loci. (DOCX 19 kb)
