# Supplementary material for: Aedes aegypti has spatially structured and seasonally stable populations in Yogyakarta, Indonesia
Source: Parasit Vectors. 2015 Dec 1;8:610. doi: 10.1186/s13071-015-1230-6 (PMC4666043; doi:10.1186/s13071-015-1230-6)
Supplement: Additional file 1: Table S1. — Pairwise F ST comparisons between Aedes aegypti adults and larvae. Individuals collected in wet season were scored at nine microsatellite loci. Table S2. STRUCTURE Harvester results for adult and larval mosquitoes that were scored at nine microsatellite loci. (DOCX 19 kb) [file 13071_2015_1230_MOESM1_ESM.docx]

Table S1. Pairwise comparisons of genetic distance between adults and larvae of *Aedes aegypti* at six sites in Indonesia in Season 1 where bold indicates significance at the table-wide α = 0.05 level.

| **Adults *vs*. larvae** | ***F*_ST_** | ***P*-value** |
| --- | --- | --- |
| Site 01 | -0.0001 | 0.6667 |
| Site 02 | -0.00215 | 0.7387 |
| Site 03 | 0.00293 | 0.40541 |
| Site 05 | -0.00387 | 0.75676 |
| Site 06 | 0.02723 | **0.00901** |
| Site 10 | 0.00416 | 0.31532 |

Table S2. STRUCTURE Harvester results for the adult and larval mosquitoes scored at nine micorsatellite loci.

| Adults season 1 |  |  |  |  |  |  |
| --- | --- | --- | --- | --- | --- | --- |
| # K | Reps | Mean LnP(K) | Stdev LnP(K) | Ln'(K) | \|Ln''(K)\| | Delta K |
| 1 | 5 | -3117.86 | 0.34 | NA | NA | NA |
| 2 | 5 | -3052.76 | 4.09 | 65.10 | 21.74 | 5.31 |
| 3 | 5 | -3009.40 | 12.61 | 43.36 | 35.74 | 2.83 |
| 4 | 5 | -3001.78 | 30.35 | 7.62 | 35.66 | 1.17 |
| 5 | 5 | -3029.82 | 38.88 | -28.04 | 15.74 | 0.40 |
| 6 | 5 | -3042.12 | 19.41 | -12.30 | NA | NA |
| Larvae season 1 |  |  |  |  |  |  |
| # K | Reps | Mean LnP(K) | Stdev LnP(K) | Ln'(K) | \|Ln''(K)\| | Delta K |
| 1 | 5 | -7464.64 | 0.05 | NA | NA | NA |
| 2 | 5 | -7377.64 | 14.39 | 87.00 | 55.74 | 3.87 |
| 3 | 5 | -7346.38 | 16.22 | 31.26 | 65.60 | 4.04 |
| 4 | 5 | -7380.72 | 45.37 | -34.34 | 21.22 | 0.47 |
| 5 | 5 | -7436.28 | 135.10 | -55.56 | 157.36 | 1.16 |
| 6 | 5 | -7334.48 | 112.90 | 101.80 | 197.08 | 1.75 |
| 7 | 5 | -7429.76 | 144.15 | -95.28 | 1.30 | 0.01 |
| 8 | 5 | -7523.74 | 300.67 | -93.98 | 40.20 | 0.13 |
| 9 | 5 | -7577.52 | 193.01 | -53.78 | 36.12 | 0.19 |
| 10 | 5 | -7667.42 | 161.58 | -89.90 | NA | NA |
| Larvae season 2 |  |  |  |  |  |  |
| # K | Reps | Mean LnP(K) | Stdev LnP(K) | Ln'(K) | \|Ln''(K)\| | Delta K |
| 1 | 5 | -6726.94 | 0.0548 | NA | NA | NA |
| 2 | 5 | -6668.52 | 9.5429 | 58.42 | 184.06 | 19.287632 |
| 3 | 5 | -6794.16 | 115.02 | -125.64 | 26.76 | 0.232655 |
| 4 | 5 | -6893.04 | 268.6453 | -98.88 | 231.96 | 0.863443 |
| 5 | 5 | -6759.96 | 67.0721 | 133.08 | 378.08 | 5.636921 |
| 6 | 5 | -7004.96 | 328.4983 | -245 | 266.34 | 0.81078 |
| 7 | 5 | -6983.62 | 121.1074 | 21.34 | 33.42 | 0.275953 |
| 8 | 5 | -6928.86 | 195.7856 | 54.76 | NA | NA |
